# Peculiarities in the panoramic radiograph of patients with secondary hyperparathyroidism due to terminal renal disease: a radiologic controlled comparative study

**DOI:** 10.1007/s11282-022-00613-2

**Published:** 2022-05-05

**Authors:** Tobias Moest, Arne Eric Jahn, Katharina Heller, Mario Schiffer, Werner Adler, Maximilian Rohde, Manuel Weber, Marco Rainer Kesting, Rainer Lutz

**Affiliations:** 1 Department of Oral and Maxillofacial Surgery, University Hospital Erlangen, Glückstraße 11, 91054 Erlangen, Germany; 2 Department of Nephrology and Hypertension, University Hospital Erlangen, Ulmenweg 18, 91054 Erlangen, Germany; 3 Department of Medical Informatics, Biometry and Epidemiology (IMBE), University of Erlangen-Nuremberg, Waldstraße 6, 91054 Erlangen, Germany

**Keywords:** Terminal renal insufficiency, Renal osteodystrophy, Secondary hyperparathyroidism, Panoramic radiograph

## Abstract

**Objectives:**

The present radiological observational controlled study aims to evaluate the impact of secondary hyperparathyroidism (SHPT) due to chronic kidney disease (CKD) as well as the duration of dialysis on the mineralization of the mandible by standardized qualitative evaluation of digital panoramic radiographs.

**Methods:**

Panoramic radiographs of CKD patients with SHPT and healthy controls were used for the qualitative analysis of the mandibular cortical index (MCI), the trabecular bone pattern (TBP), and calcification and resorption foci. Radiomorphometric indices were correlated to biochemical parameters and the duration of dialysis using the Spearman Rho test. Group comparisons were conducted using the Mann–Whitney *U* test and Fisher’s exact test at a significance level of *α* ≤ 0.05. Interrater reliability of two physicians was estimated using Cohen’s kappa.

**Results:**

Inclusion and exclusion criteria were fulfilled by *N* = 41 patients. Statistically significant differences in the MCI (*p* < 0.001) as well as the TBP (*p* = 0.002) could be detected for the experimental group in comparison to the healthy control group. Focusing on calcification and resorption foci, no statistically significant difference could be detected between the groups (*p* = 0.244). The level of the detected parathyroid hormone (PTH) significantly correlated with TBP (Rho = 0.338; *p* = 0.031), while no significant relationship between TBP and the duration of the dialysis could be found.

**Conclusions:**

Patients with SHPT due to CKD show statistically significant bone changes in the panoramic radiograph, whereby the grade of trabecular bone change correlates to PTH values.

## Introduction

In 2016, chronic kidney disease (CKD) affected 753 million people globally [1]. Epidemiological data suggest that renal insufficiency has an increasing global relevance. In 2015, CKD caused 1.2 million deaths compared to “only” 409,000 in 1990 [2, 3]. The most common causes of CKD are diabetes mellitus, hypertension, and glomerulonephritis [4].

The clinical symptoms and signs are varied, with increased blood pressure, accumulation of urea, hyperkalemia, fluid overload, metabolic acidosis, reduced erythropoietin production leading to anemia, and changes in mineral and bone metabolism resulting from decreased renal function. Changes in the calcium and phosphate balance can be explained by the decreased endocrine function due to the insufficient activation of vitamin D into its active form and the inadequate excretion of phosphate. As a result of the phosphate overload, insoluble calcium phosphate forms, removing calcium from the circulation and subsequently leading to hypocalcemia. This hypocalcemia then activates the parathyroid gland, leading to excessive secretion of parathyroid hormone (PTH) (secondary hyperparathyroidism; SHPT) to increase serum calcium levels again.

CKD is the most common cause of SHPT followed by different causes of malabsorption that are related to the reduced absorption of fat-soluble vitamin D. PTH releases calcium from bones by activating osteoclasts via the receptor activator of nuclear factor-kappa B (RANK) and its ligand (RANKL) system. At the same time, PTH promotes the reabsorption of calcium from the kidney. Although phosphate is also released from the bones under the influence of PTH, its excretion via the kidney is increased, so that the overall phosphate serum level decreases. The disturbances in the serum levels of calcium, phosphate, PTH, and vitamin D and their effects on bone turnover, mineralization, and extra-skeletal calcifications are summarized under the term “renal osteodystrophy.”

Renal osteodystrophy thus represents an osseous disease that results in decreased bone density manifesting in osteoporosis, osteopenia, or osteomalacia. As a result, skeletal abnormalities such as trabecular bone alterations (ground glass appearance), subperiosteal resorption, and osteitis fibrosa cystica, also known as “brown tumors,” can be found in radiological dental imaging [5–8]. In this context, panoramic radiographs could provide an additional tool for diagnosing and monitoring bone/bone mineral loss in CKD patients, as there is ample evidence that a number of indices of the panoramic radiograph are suitable for detecting initial bone loss [9]. In particular, Kinalski et al. demonstrated that, due to its high sensitivity, the mandibular cortical index (MCI) is an adequate tool for the screening of initial bone mineral density loss in women over 30 years of age [9]. The analysis of panoramic radiographs of CKD patients with SHPT has already been described in literature [10–12]. The informative value about its diagnostic potential is, however, limited due to the selection of inclusion criteria. In their 2019 study, Queiroz et al., for example, included postmenopausal women in their analysis. Here, the interpretation of the results may be limited by the fact that this group suffers from impaired bone mineralization due to primary osteoporosis [12]. Due to this fact, only male patients should be included in radiological data analysis to prevent measuring the effect of primary osteoporosis in postmenopausal women. Henriques et al. included only male patients with a minimum age of 45 years, PTH levels of ≥ 500 pg/ml, and hemodialysis duration of at least three years in their study [10]. This cohort represents a patient group where a high loss of bone minerals with corresponding changes in the panoramic radiograph is to be expected. Unfortunately, due to the selected inclusion and exclusion criteria, no statement concerning osseous changes in the panoramic radiograph for male patients with pathologic PTH levels (> 65 pg/ml) under 500 pg/ml with less than three years of dialysis could be given.

Panoramic radiographs are quick, cheap, and easy to perform and are associated with a low radiation dose, and thus represent a standard diagnostic tool in everyday dental and oral/maxillofacial practice. Virtually every patient entering a dentist’s/surgeon’s office with a dental-related problem will receive a panoramic radiograph. With reliable expansion of the indication range of the panoramic radiograph, the attending dentist has the opportunity to contribute to the diagnosis and screening of SHPT and its associated bone changes.

Building on the methods of Henriques et al., the aim of the present study is to investigate and demonstrate the reliability and the diagnostic potential of panoramic radiography parameters with respect to the visualization/representation of osseous changes of the mandible in a sample of patients with advanced CKD and different stages of SHPT. Furthermore, the correlation between PTH levels, duration of dialysis, and panoramic radiograph parameters is to be analyzed.

## Materials and methods

### Study characteristics

The observational radiological case control study was performed at the Erlangen University Hospital Department of Oral and Maxillofacial Surgery in cooperation with the Department of Nephrology and Hypertension. Dialysis patients with CKD presented in the Department of Oral and Maxillofacial Surgery for clinical and radiological intraoral health assessment prior to organ transplantation as a part of the organ transplantation listing process.

Based on the age distribution of the experimental group, patients were selected from the radiological data pool who did not have any osseous diseases or medications that had an impact on the osseous mineralization.

The study covers an observation period from January 2015 to March 2020.

Ethical approval (Petition No. 450_20Bc) was obtained from the ethics committee of the medical faculties of the Friedrich-Alexander-Universität Erlangen-Nürnberg.

### Inclusion and exclusion criteria

Male CKD patients who were undergoing hemodialysis at a kidney center were selected according to the following inclusion and exclusion criteria: Stage V of CKD (glomerular filtration rate < 15 ml/min/1.73 m^2^), hemodialysis with associated secured diagnosis of SHPT, pathologic PTH levels of 65 > pg/ml. Patients with a confirmed SHPT but PTH levels within or lower than the reference (due to parathyroidectomy) were excluded from the data analysis.

### Outcome variables and hypothesis

The primary outcome variable of the study was to quantify the radiological indices of the mandible in patients with SHPT due to CKD by standardized qualitative evaluation of panoramic radiographs.

The secondary outcome variable was to verify whether the degree of SHPT (PTH level) or the duration of hemodialysis correlates with the degree of bone mass/density.

As a null hypothesis, it is assumed that regardless of the duration of the dialysis and the PTH levels, no difference concerning the panoramic radiography parameters can be detected when comparing the dialyzed CKD group with the healthy control group.

### Radiological analysis and measurements

Standard panoramic digital radiographs (Sirona Orthophos XG; settings: acquisition time = 14.1 s., 64 kV, 16 mA; Sirona Dental Systems GmbH, Bensheim, Germany) were performed in the Department of Oral and Maxillofacial Surgery of the University Clinic Erlangen following the manufacturer’s positioning and exposure protocol as part of a routine examination. The radiographs were made available in the SIDEXIS XG database and software and saved for further analysis in TIFF format.

**Fig. 1:**
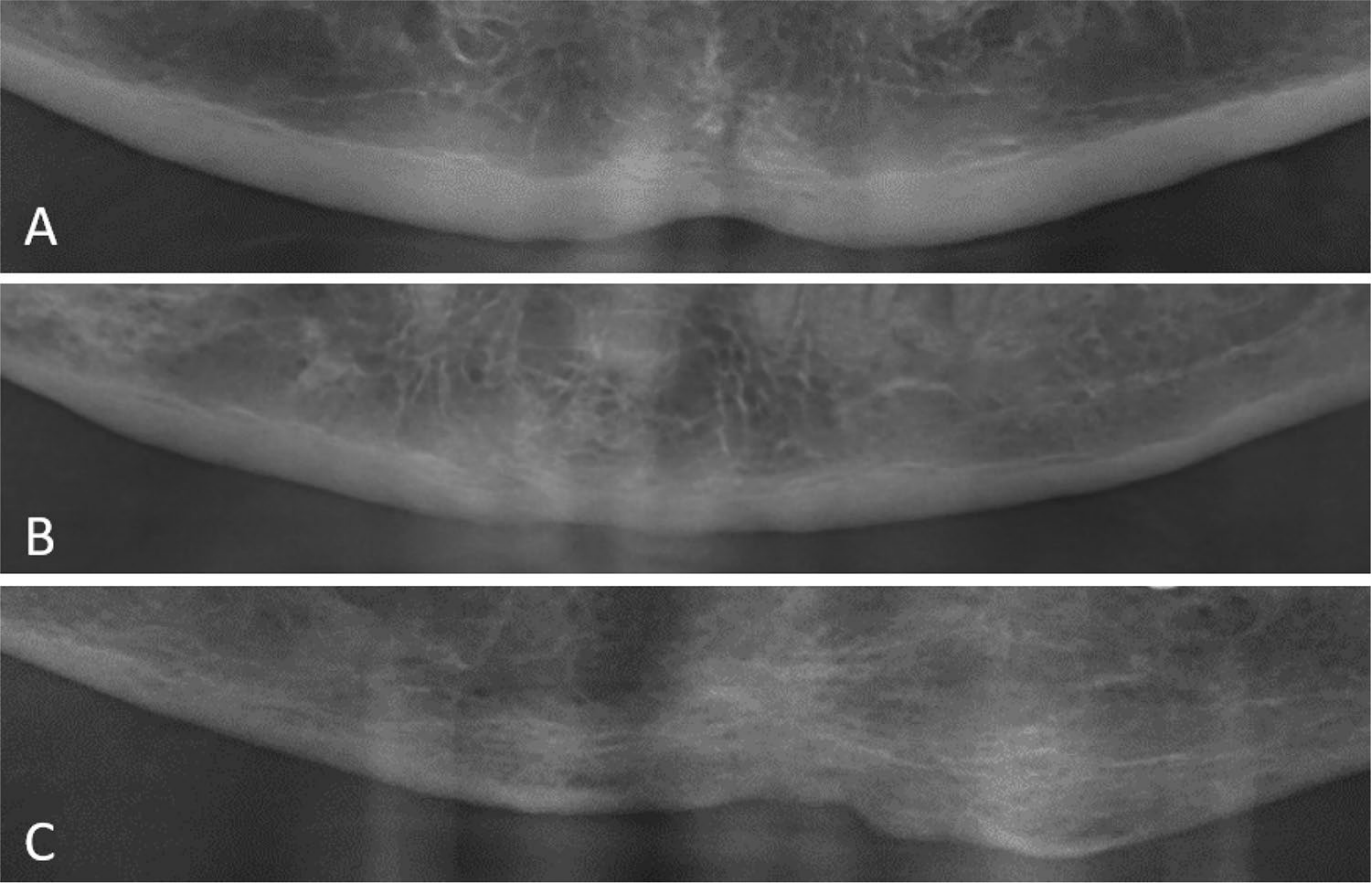
Exemplary demonstration of anterior mandible segments of standardized panoramic radiographs. **A** shows the morphological appearance of “C1” (normal cortex), **B** illustrates “C2” (moderately eroded cortex), and **C** demonstrates “C3” (severely eroded cortex) cortical bone quality

**Fig. 2:**
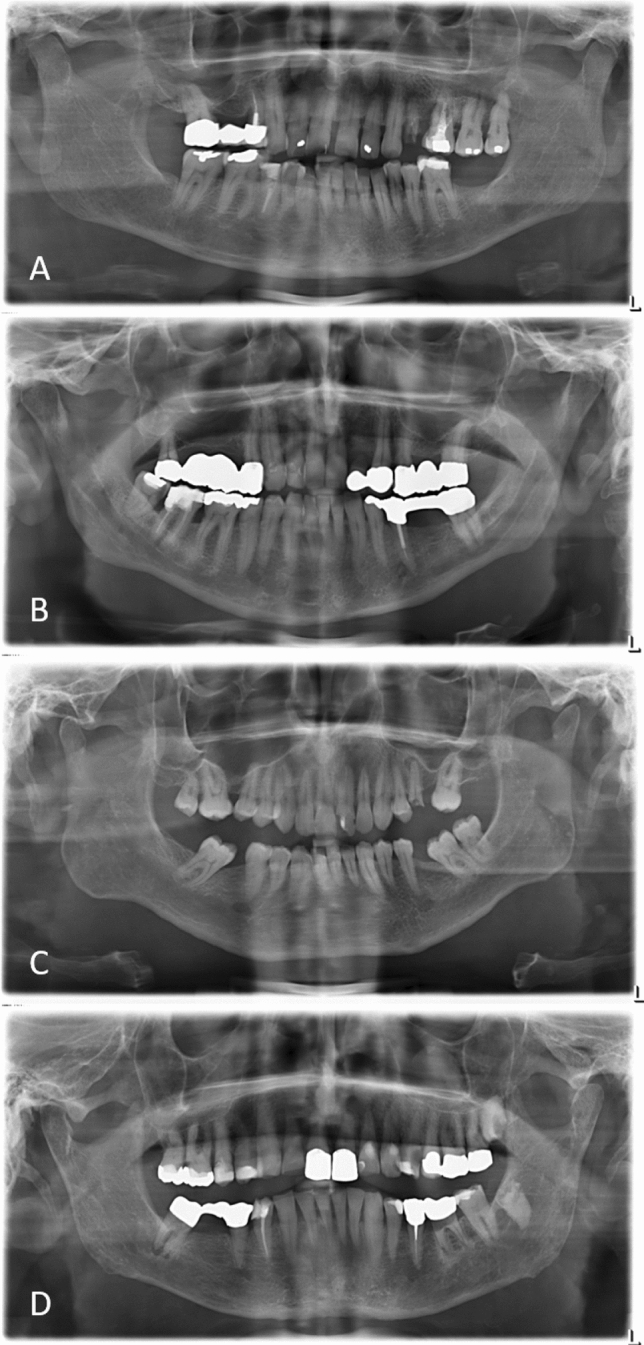
Exemplary demonstration of panoramic radiographs showing dense (**A**), heterogenous (**B**), sparse (**C**), and sparse with ground glass appearance (**D**) mandibular bone quality

**Fig. 3:**
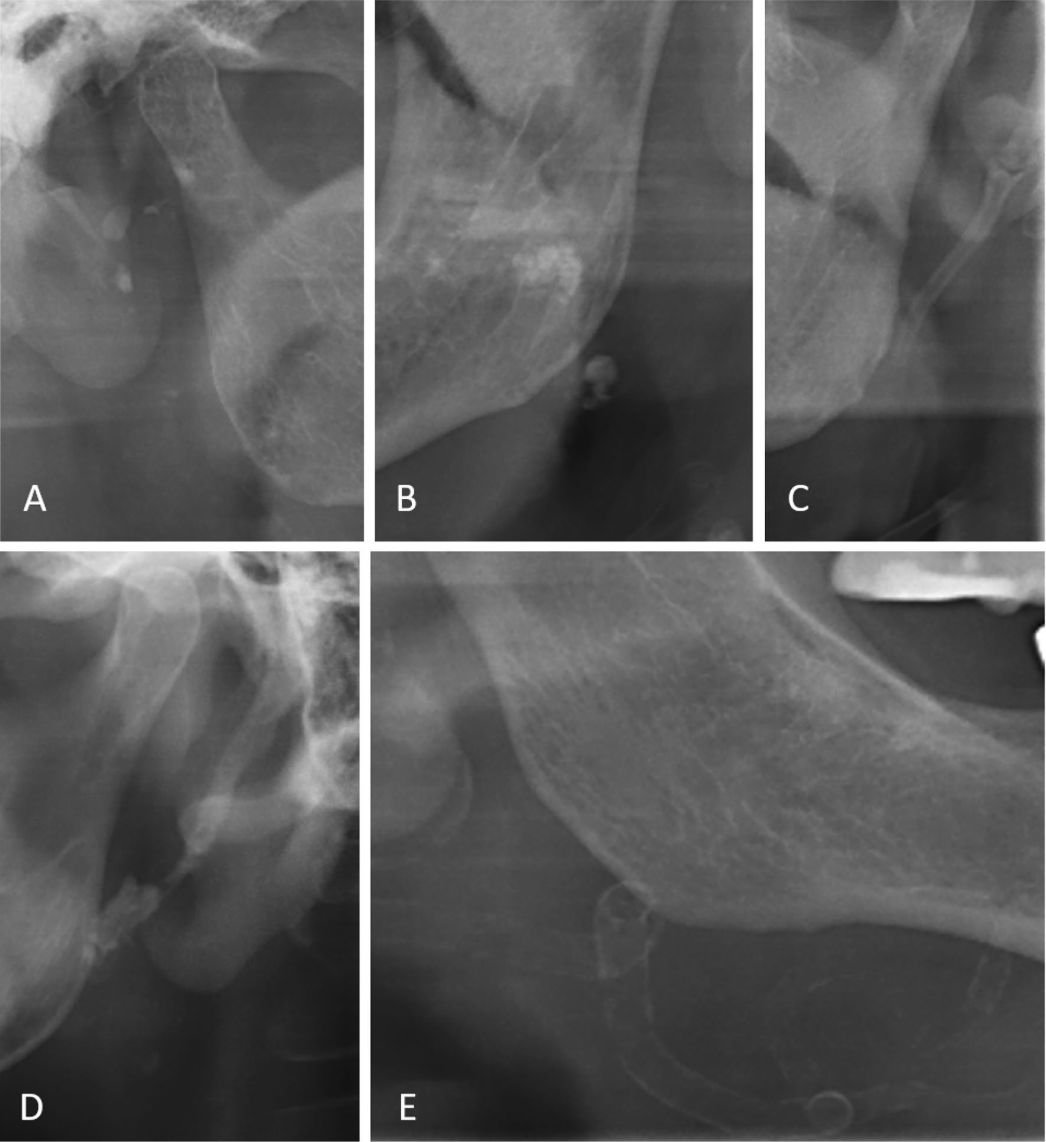
Exemplary demonstration of different calcification foci: parotid gland (**A**), tonsillar region (**B**), ligamentum stylohyoideum (**C** + **D**), and the facial artery (**E**)

Building on the aforementioned evaluation methods of Henriques et al. for radiological analysis of the digital panoramic radiographs for signs of osseous changes, the following parameters were selected [10]:

1. Mandibular cortical index (MCI, qualitative parameter): morphological appearance of the mandibular cortical bone distally to the mental foramen by bilateral inspection; “C1” represents normal cortex, “C2” moderately eroded cortex, and “C3” severely eroded cortex [13] (Fig. 1).
2. Trabecular bone pattern (TBP, qualitative parameter): morphological appearance of the trabecular bone in the mandibular body by bilateral inspection: “D” for dense, “H” for heterogenous, “S” for sparse, and “SwGGA” for sparse with ground glass appearance [10, 14] (Fig. 2).
3. Calcification and resorption foci (“CaR,” qualitative parameter) (Fig. 3).

The measurements and analyses of the data were performed by two evaluators: an experienced resident for oral and maxillofacial surgery and an experienced oral and maxillofacial surgeon who has worked in radiological training for dental students for more than six years and as a radiation protection officer.

Both evaluators were trained for 2 weeks in the application of the mentioned qualitative parameters using 20 panoramic radiographs: 10 from dialyzed CKD patients and 10 from healthy controls. Divergent results were discussed to increase reliability. After calibration, data were analyzed in a double-blind design with an interval of 14 days between the first and second assessment. Intra- and interobserver agreement was tested using the kappa index for qualitative parameters (mandibular cortical index and trabecular bone pattern).

### Biochemical analysis

In several CKD patients, the biochemical parameters and dosages of PTH, calcium (Ca), phosphate (P), and Ca x *P* were obtained by the Department of Nephrology and Hypertension to verify the diagnosis and cluster the investigation groups by the expression of SHPT.

### Statistical analysis

For interrater reliability, the kappa index was used with a one-week interval showing substantial agreement. Fisher’s exact test was used to determine statistical differences between the experimental dialyzing and the healthy control groups. A *P* value of less than 0.05 was considered to be significant. The statistical analysis was performed using the publicly available statistical software “R” V3.6.3 [15].

## Results

### Interrater agreement

For the analyses of MCI (kappa = 0.565 (95% CI 0.404; 0.726; *p* < 0.001), TBP (kappa = 0.765 (95% CI 0.647; 0.883; *p* < 0.001) as well as the calcification/resorption foci (kappa = 1 (95% CI 1; 1; *p* < 0.001), the intraclass correlation coefficient indicated excellent interrater agreement.

### Characterization of study population

From a total of *N* = 51 patients, ten patients fulfilled the exclusion criteria, thus the defined inclusion criteria were fulfilled by *N* = 41 patients. Mean age of included patients was 53.3 ± 13.0 years, whereby the youngest patient was 22 years and the oldest was 79 years old. Mean PTH level was 361.9 ± 316.1 pg/ml, with a minimum level of 69.2 pg/ml and a maximum level of 1299.0 pg/ml. The mean Ca value was 2.3 ± 0.2 mmol/l (minimum: 1.9 mmol/l; maximum: 2.6 mmol/l). Mean *P* value was 1.7 ± 0.4 mmol/l (minimum: 0.8 mmol/l, maximum 2.9 mmol/l). The mean Ca x *P* value showed 3.9 ± 1.0 mmol/l (minimum: 1.6 mmol/l, maximum 6.8 mmol/l). Mean duration of dialysis was 1054.4 days (± 1016.6 days) with a minimal duration period of 25 days and a maximum period of 4028 days.

### Comparison of qualitative parameters between CKD patients and healthy controls

**Fig. 4:**
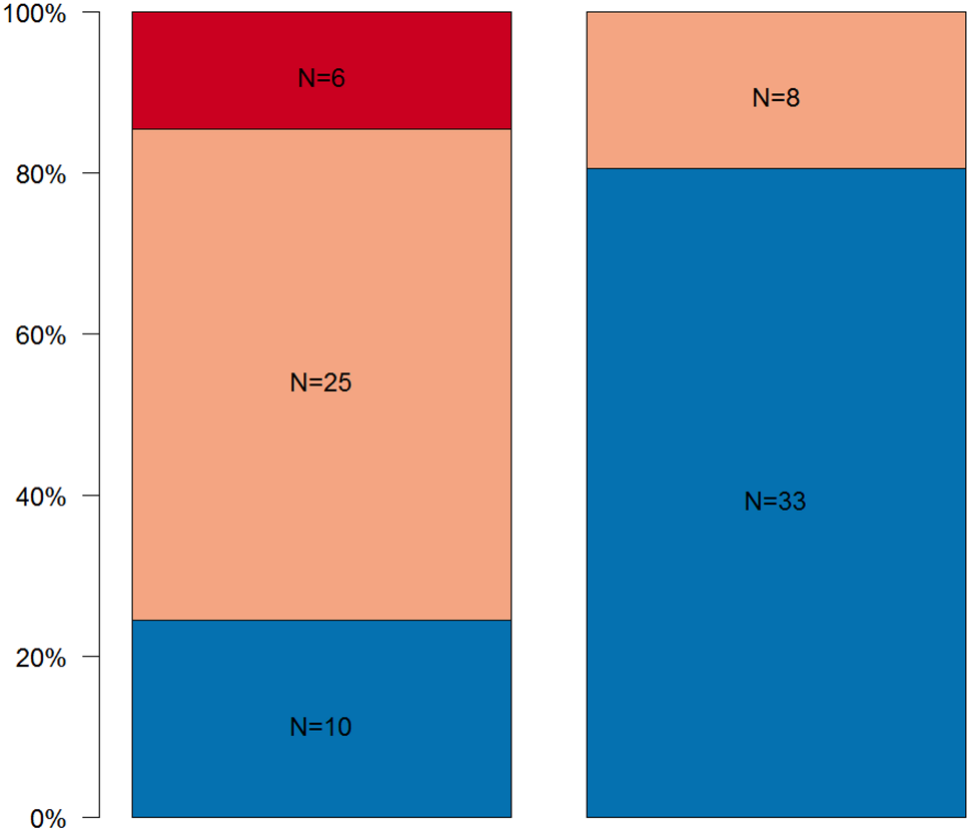
Distribution of panoramic findings using the MCI for the experimental group (diagnosis of SHPT with pathologic PTH levels) (left column) and the healthy control group (right column). Blue represents normal cortex, orange stands for moderately eroded cortex, and red means severely eroded cortex

**Fig. 5:**
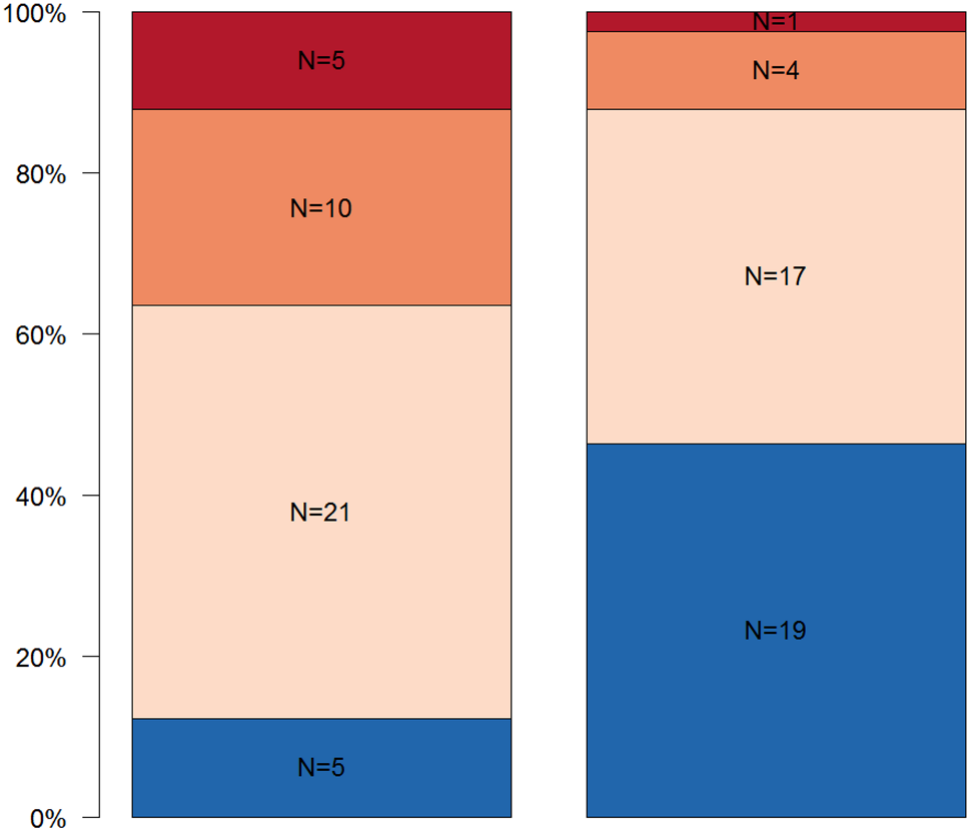
Distribution of panoramic findings describing the trabecular bone pattern for the experimental group (diagnosis of SHPT with pathologic PTH levels) (left column) and the healthy control group (right column). Blue represents dense, bright-orange stands for heterogenous, orange means sparse, and red means sparse with ground glass trabecular bone appearance

The results of the qualitative inter-group comparisons are summarized in Figs. 4, 5. Regarding the MCI, statistically significant differences within the experimental dialyzing group and healthy control group (*p* < 0.001) could be found. In the dialyzing group *N* = 10 (24.4%) men showed a normal cortex structure, *N* = 25 (61.0%) men a moderately eroded cortex, and *N* = 6 (14.6%) patients a severely eroded mandibular cortex structure. In the healthy control group, in *N* = 33 (80.5%) patients a normal cortical structure and in *N* = 8 (19.5%) men a moderately eroded cortex structure could be detected (Fig. 4).

Focusing on the TBP index, a statistically significant difference could be detected between the experimental dialyzing group and the healthy control group (*p* = 0.002). In the experimental dialyzing group, *N* = 5 (12.2%) patients showed dense bone, *N* = 21 (51.2%) patients heterogenous bone, *N* = 10 (24.4%) sparse bone, and in *N* = 5 (12.2%) patients sparse bone with ground glass appearance. In the healthy control group, *N* = 19 (46.3%) patients showed dense bone, *N* = 17 (41.5%) patients heterogenous bone, *N* = 4 (9.8%) sparse bone, and *N* = 1 (2.4%) patients sparse bone with ground glass appearance (Fig. 5).

In patients of the experimental group, more CaR foci (experimental *N* = 30 (73.2%) patients vs. healthy control *N* = 24 (58.5%) patients) could be found. The difference between the two groups was not statistically significant (*p* = 0.244). In Fig. 3 ectopic calcifications are illustrated in the region of the parotid gland (A), tonsillar region (B), ligamentum stylohyoideum (C + D), and the facial artery (E).

### Correlation of PTH levels with the panoramic radiography parameters of CKD patients

Regarding the correlation of the diagnosed PTH levels and the prevalence of ectopic calcifications, no statistically significant correlation could be proven (Rho = 0.037; *p* = 0.817). The same is true for the MCI. Here, no statistically significant correlation exists between the PTH levels and the grade of cortex erosion (Rho = 0.165; *p* = 0.302). However, the grade of the detected PTH levels correlates statistically significantly with TBP (Rho = 0.338; *p* = 0.031).

### Relationship between dialysis duration and the panoramic radiography parameters of CKD patients

Duration of dialysis did not differ significantly between patients with etopic calcification and those without etopic calcification (Mann–Whitney *U* test: *p* = 0.233). Furthermore, the dialysis duration did not differ significantly between different MCIs (Kruskal–Wallis test: *p* = 0.100). Moreover, no statistically significant connection of the TBP and dialysis duration could be found (Kruskal–Wallis test: *p* = 0.565).

## Discussion

Dentistry is increasingly specializing in the fields of oral surgery, preservation, periodontology, prosthodontics, and orthodontics. Nevertheless, all disciplines require knowledge and consideration of anamnestic conditions, such as the presence of underlying internal diseases, the associated long-term medication, and their influence on the stomatognathic system.

Particularly in patients suffering from chronic renal failure, there are a number of internal and pharmacological peculiarities that the practitioner must be aware of. The physician should be able to recognize that, resulting from renal failure and due to an accumulation of urinary substances, in addition to fatigue, loss of appetite, and headache, a “foetor ex ore” can occur as a typical sign of uremia [16]. Furthermore, surgeons should be aware of hemolysis resulting from uremia toxins (anemia), thrombopenia, or thrombocyte and leukocyte dysfunction in patients with CKD [17–19]. Further endocrine consequences of renal insufficiency entail normochromic, normocytic anemia due to erythropoietin deficiency as well as a change in bone metabolism (renal osteopathy) due to hyperphosphatemia and hypocalcemia in the absence of calcitriol, leading to secondary hyperparathyroidism with increased parathyroid hormone levels.

In this context, in their 2014 study, Henriques et al. were able to show that a change in bone metabolism due to chronic renal insufficiency can be detected in dental X-ray diagnostics in male patients with severe secondary hyperparathyroism (PTH levels of ≥ 500 pg/ml) and a dialysis period of at least three years [10]. However, the influence of pathologic PTH levels < 500 pg/ml and a dialysis duration of less than three years remains unanswered, which means that further investigations in this area are necessary. Based on this data, the aim of the current study was to determine the diagnostic potential of dental X-ray diagnostics by means of panoramic radiographs also for patients with PTH levels < 500 pg/ml and a dialysis duration of less than 3 years.

To minimize the risk of misinterpretation of the generated data, strict inclusion and exclusion criteria were defined. The patient group was characterized by the confirmed diagnosis of SHPT within known chronic terminal renal failure and the requirement for dialysis. Due to the risk of including the effect of primary osteoporosis due to estrogen deficiency in postmenopausal women, only male patients were included in the data analysis. Patients who had parathyroid levels within or lower than the reference range (due to parathyroidectomy) despite confirmed SHPT at the time of presentation in our department were also not included in the data analysis.

When looking at the results of the present study, the panoramic radiograph represents a potentially suitable method to diagnose osseous changes resulting from secondary hyperparathyroidism. Especially for MCI and TBP statistically significant differences between the patient group and the control group could be detected.

However, significantly increasing cortical erosion in combination with a reduced TBP was expected to occur with increasing PTH levels, but not present in the patient collective. Nevertheless, it must be stated that the radiological findings/parameters do not perfectly correlate with the measured PTH levels, since the MCI and the TBP were not affected in the same way by pathologic PTH levels. Our data show that the cortical mandibular PTH-induced erosion is less pronounced than the change in the trabecular bone patterns. There was a positive and statistically significant correlation between PTH levels and the grade of TBP changes. The MCI changes did however not correlate with PTH. These results seem to be understandable, since the mineralization rate of the cortical bone is superior compared to the cancellous bone, whereby the “loosening” effect of the PTH is delayed noticeably in the cortical aspects. In this context, PTH levels of > 400 pg/ml seem to have a significant impact on the TBP.

Another unexpected effect was that the duration of the dialysis did not significantly correlate with MCI or the TBP, which speaks for the effective dialysis in combination with medication/substitution.

The evaluation of the absorption rate or ectopic calcification rate between the experimental and the control group also showed unexpected results. Here, Henriques et al. were able to show that patients with SHPT suffer significantly more frequently from absorption or mineralization disorders compared to healthy control patients [10]. For this reason, we would also have expected an increased incidence of mineralization disorders in patients with SHPT. However, the statistical analysis showed no statistically significant difference between the control group and the dialyzing experimental group. Furthermore, within the dialyzing experimental group, no correlation between the levels of PTH and the frequency of mineralization disorders was found.

From a pathophysiological point of view and as mentioned before, SHPT leads to an imbalance of calcium and phosphate in the blood, resulting in changes to the mineral metabolism and mineral deposition in the form of tonsilloliths, calcified lymph nodes, or ossification of the styloid ligaments. However, we found that those patients with SHPT who had ossifications had this particularly pronounced. In some patients, the mineralization was so pronounced that it could be detected in the vessels as atherosclerosis (Fig. 3E), which as a cardiovascular disease represents the main cause of death in this population [20, 21]. With the radiological experience to recognize these findings in combination with the necessary internal medicine experience, dentists represent an essential aid to patient education and sensitization to prophylaxis to extend lifetime. Although in case of an accidental detection of atherosclerotic plaque in a cervical vessel direct prophylaxis is not completely possible by a dentist, the dentist could enter a new competence area which addresses raising the patient’s awareness of the potential disease as well as the initiation of further diagnostic (e.g., imaging diagnostics like ultrasound diagnostics) and therapeutic steps by consultation with a general practitioner.

As a possible study limitation, it must be mentioned that due to the selected inclusion and exclusion criteria the number of included patients (*N* = 41) is relatively low. Furthermore, it must be stated that the selected study design includes just one evaluation time point. The changes over time of the determination of mineralization of the mandible depending on changing renal function and the duration of dialysis would be interesting and could be the aim of further prospective studies.

## Conclusions

The evaluation of the panoramic radiograph allows the detection of osseous changes of the lower jaw in patients with SHPT due to CKD. In this context trabecular bone change correlates to PTH values.
